# A Versatile Fabrication Route for Screening of Block Copolymer Membranes in Bioprocessing

**DOI:** 10.1021/acsomega.4c11269

**Published:** 2025-02-20

**Authors:** Ke Meng, Alberto Alvarez-Fernandez, Stefan Guldin, Daniel G. Bracewell

**Affiliations:** 1 Department of Biochemical Engineering, University College London, Gower Street, London WC1E 6BT, U.K.; 2 Centro de Fisica de Materiales (CFM)(CSICUPV/EHU), Materials Physics Center (MPC), San Sebastian 20018, Spain; 3 Department of Chemical Engineering, University College London, Torrington Place, London WC1E 7HB, U.K.; 4 Department of Life Science Engineering, Technical University of Munich, Gregor Mendel-Straße 4, Freising 85354, Germany; 5 TUMCREATE, 1 CREATE Way, #10-02 CREATE Tower, Singapore 138602, Singapore

## Abstract

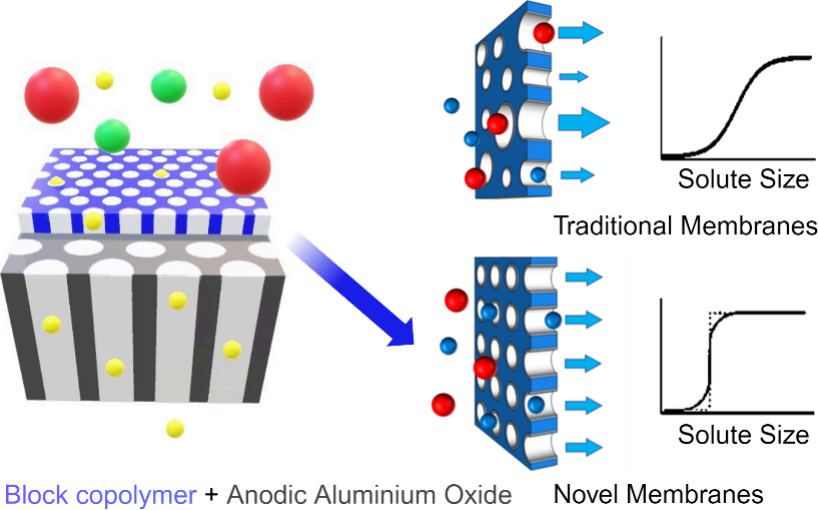

Traditional poly(ether sulfone) (PES) filters, widely used for sterile, viral, and ultrafiltration, often exhibit restrictions in their selectivity-permeability profile due to their heterogeneous pore size distribution. This limitation has sparked interest in developing novel isoporous membrane materials and fabrication techniques. Among promising candidates, block copolymer (BCP) membranes produced via self-assembly and nonsolvent-induced phase separation (SNIPS) offer significant advantages, including tunable pore size, narrow pore size distribution, high porosity, and enhanced mechanical flexibility. However, optimizing the structure formation in SNIPS remains a complex and time-consuming process, making it unsuitable for rapidly screening new BCP candidates. In response, this study introduces an alternative fabrication approach based on the direct spin-coating of BCPs onto anodic aluminum oxide (AAO) supports. Using this method, a poly(styrene)-*block*-poly(methyl methacrylate) (PS-*b*-PMMA) thin film was directly cast onto a water-filled AAO support, enabling the formation of an isoporous membrane structure for filtration applications, significantly reducing the complexity of structure–application optimization. When compared to commercial PES membranes with similar molecular weight cut-offs, these novel PS-*b*-PMMA thin-film composite membranes exhibited comparable transmission rates for bovine serum albumin and a monoclonal antibody, while delivering a ninefold improvement for thyroglobulin rejection. This superior cutoff precession highlights their potential to remove viruses and antibody aggregates during the downstream processing of monoclonal antibody production. By reducing the burden of chromatographic polishing steps, this advance offers promise for enhancing efficiency and lowering costs in biopharmaceutical manufacturing.

## 1 Introduction

Genomic medicines based on viral and nonviral vectors are transforming our approach to treating cancer, viral infections and genetic disorders. Despite their promise, the unique characteristics of these vectors -such as their size, stability, and the need for precise product-related impurities removal- pose significant challenges in downstream processing.^1^ Similar challenges are found in antibody manufacture, a more mature market projected to generate $ 300 billion by 2025,^2^ with additional growth anticipated from emerging therapies for illnesses such as dementia and other neurodegenerative diseases.^3^ Critical to all these biomanufacturing processes are membrane-based unit operations like ultrafiltration, and virus and sterile filtration. However, these processes often face limitations due to the low throughput caused by membrane fouling and the rigorous requirements for impurity clearance.^4^

In this context, evaluating membrane performance requires a comprehensive analysis based on several key parameters, including selectivity, permeability, mechanical integrity, and antifouling properties. Each of these factors plays a vital role in determining the overall effectiveness of the membrane in specific separation challenges.^5^ Among these parameters, selectivity and permeability are particularly critical, often exhibiting a “trade-off” relationship in commercial ultrafiltration membranes, similar to the well-known Robeson Plot for gas separation membranes.^6,7^ The relationship between selectivity and permeability in commercial ultrafiltration membranes was initially explored by Mehta and Zydney in 2005.^8^ Since then, comparisons of commercial membranes have established an ‘upper bound,’ setting the benchmark for leading performance standards in the field.

Following this classification, poly(ether sulfone) (PES)-based membranes have been the state-of-the-art in membrane technology for several decades due to their versatility and scalability.^9−11^ This adaptability is largely attributed to the fabrication method employed, with these membranes typically manufactured using the well-established nonsolvent induced phase separation (NIPS) method, pioneered by Loeb and Sourirajan in 1963.^12^ However, despite their extensive applications, PES membranes still present challenges, particularly regarding their selectivity. This issue, largely attributed to the wide range of pore sizes in the surface separation layer, can significantly impact the efficiency of the filtration process, often leading to product losses or extended processing times, making them unsuitable for biomedical applications, which often involves working with low quantity samples.^13^ Therefore, there has been a strong research effort focused on the development of innovative materials and fabrication techniques to overcome these limitations.^14,15^

From a material perspective, a variety of novel candidate materials have been explored, including self-assembled block copolymers (BCPs),^16−18^ anodic aluminum oxide (AAO),^19−21^ photoresist polymers,^22^ porous silicon,^23^ and carbon nanotubes.^24^ All these materials share the potential to achieve uniform pore size distributions, high porosity, and straight pore channels, which are essential for optimizing selectivity-permeability performance.^25^ Among these options, BCP-based membranes offer unique advantages that have garnered significant attention.^26^ Their formation principles, based on molecular self-assembly, enable the creation of membranes with customizable pore sizes across a broad spectrum, narrower pore dispersity, and high porosity by controlling parameters such as BCP molecular weight, the volume fraction between the blocks and the BCP polydispersity index.^16^ Additionally, their polymeric nature allows for easy integration into various filtration modules, and offers superior mechanical properties compared to their inorganic counterparts, thereby expanding their applicability.^27,28^

Thus, different BCP systems, such as poly(styrene)-*block*-poly(methyl methacrylate) (PS-*b*-PMMA), poly(styrene)-*block*-poly(ethylene oxide) (PS-*b*-PEO),^29^ poly(styrene)-*block*-poly(vinylpyridine) (PS-*b*-PVP),^30^ and more complex tert- and tetra-BCPs,^31,32^ have been successfully employed for the fabrication of tailored and highly homogeneous isoporous membranes. The rationale behind selecting these systems lies in their high incompatibility between the blocks, which facilitates successful microphase segregation and the formation of well-defined nanostructures; the differences in reactivity between the blocks, which enable selective degradation and the formation of a porous structure; and the ability to easily modify the dimensions of the resulting nanostructures through methods such as supramolecular assembly or selective swelling, leading to highly tunable membranes.^33^

Focusing on the fabrication methodologies used for the preparation of BCP-based membranes, a wide variety of techniques have been explored, among which the previously mentioned SNIPS method received particular interest.^17,29,30^ Following this methodology, BCPs are first dissolved in a suitable solvent and then cast onto a substrate. After that, the membrane is immediately immersed in a nonsolvent, which induces phase separation, resulting in a membrane with well-defined and uniform pores. SNIPS-based BCP membranes have demonstrated promising potential for scale-up,^34,35^ as evidenced by the emergence of startups like Terapore Tech, which has successfully launched several lab-scale virus filters to the market.^36^

Nevertheless, the successful application of the SNIPS technique retains challenges, in particular around the integration of BCP self-assembly with the NIPS process.^13,37^ Careful optimization of both equilibrium and nonequilibrium thermodynamic processes is necessary to achieve a precisely defined membrane with a “honeycomb-like” separation layer and a “sponge-like” supporting structure.^36^ While SNIPS is well-established for membrane fabrication using well-known BCP candidates, the complexity of optimization makes rapid screening of new materials or molecular weight combinations challenging and time-consuming. Additionally, the material demand is significant, as the entire membrane architecture is made out of BCP, providing high demands on the synthesis and purification of novel macromolecular candidates.

Alternative BCP-based fabrication methodologies are based on thin film deposition and subsequent selective etching of one of the blocks -via physical or chemical etching,^5,13,38^ or the selective swelling of one of the blocks to generate the porous structure.^39−41^ Thin-film composite (TFC) membranes typically consist of two distinct layers: the top layer is a thin BCP film porous film (usually less than 400 nm) created via spin coating, and the subsequent annealing, floating, and selective etching. This thin layer facilitates faster mass transport while maintaining selectivity. The underlying layer often comprises a support material, such as a polymeric or ceramic substrate, which provides mechanical strength and stability to the membrane. While this fabrication route generates mesoporous thin film composite membranes with a well-defined separation layer, reproducibility and scalability remain challenging.^42,43^ One critical technical issue is the delamination of the active layer from its support. Additionally, defects often arise during the floating operation due to the poor mechanical strength of the BCP thin film. Additionally, the requirement of harmful and dangerous chemical products such as hydrofluoric acid in this step also introduces safety concerns for the personnel involved. Some mitigation strategies such as the “salt plate transfer method” and “direct spin-coating” have been proposed and developed.^31,32,44^ However, the application of these methodologies in the fabrication of membranes suitable for bioseparation processes is still limited. A reliable route for the fabrication of thin-film composite membranes remains sought after, in particular for initial BCP screening and evaluation during early stage research and development.

In response, this work presents a straightforward fabrication methodology utilizing the direct spin-coating method to deposit a PS-*b*-PMMA thin film onto an AAO microfiltration disc with an average pore size of 0.2 μm as the substrate. AAO discs offer several advantages: (a) they are hydrophilic, facilitating easy wetting of the pores; (b) they have flat and smooth surfaces, which promote the coating of a homogeneous thin film; (c) they possess high porosity, ensuring high permeability; and (d) they are rigid and incompressible during filtration. To prevent BCP from infiltrating the substrate, the AAO discs were prewetted with Milli-Q water. This allows the formation of a thin active BCP layer with controllable and homogeneous thickness, backed by a supportive and permeable AAO substrate. Various feedstocks with a wide range of hydrodynamic sizes were used to determine the filtration performance of the created TFC membrane, including fluorescein isothiocyanate-dextran (FITC-dextran), bovine serum albumin (BSA), monoclonal antibody (mAb), thyroglobulin (Tg), and latex nanoparticles (NPs) to mimic impurities, products, and viral particles in the bioseparation processes. Compared to the SNIPS methodology, the approach presented here simplifies material demands by decoupling the self-assembled surface layer from the supporting structure. This enables the rapid screening of BCP-based membranes while maintaining comparable structural features to those produced by SNIPS.

## 2 Materials and Methods

### 2.1 Materials

Block copolymer, poly(styrene)-*block*-poly(methyl methacrylate) (PS-*b*-PMMA) with a molecular weight of 180-*b*-77 kg/mol and a polydispersity index (PDI) of 1.02 was purchased from Polymer Source Inc., Canada. Anodisc aluminum oxide membranes, featuring a pore size of 200 nm and a diameter of 25 mm, were provided by Whatman (catalog no. 5140520). Biomax poly(ether sulfone) (PES) membranes with molecular weight cut-offs of 50 kDa, 100 kDa, 300 kDa, and 500 kDa, each 25 mm in diameter, were supplied by Millipore, USA. Chemical reagents included glacial acetic acid (≤99%) and toluene (anhydrous, 99.8%), were purchased from Sigma-Aldrich, UK; phosphate buffer saline (PBS) tablets were obtained from Gibco. Milli-Q water was produced using a Milli-Q Academic system by Millipore Merck. Fluorescein isothiocyanate (FITC)-dextran of molecular weights 4 kDa (FD4), 40 kDa (FD40), 150 kDa (46946), and 500 kDa (FD500S) were purchased from Merck Life Science. Unmodified polystyrene latex nanoparticles with diameters of 25 and 50 nm were acquired from Micromod GmbH, Germany, and those with a diameter of 100 nm were obtained from Merck Life Science. Thyroglobulin from bovine thyroid and bovine serum albumin were both sourced from Sigma-Aldrich, with catalogue nos. T1001 and A7030, respectively.

### 2.2 Fabrication of PS-*b*-PMMA TFC Membranes

**Figure 1:**
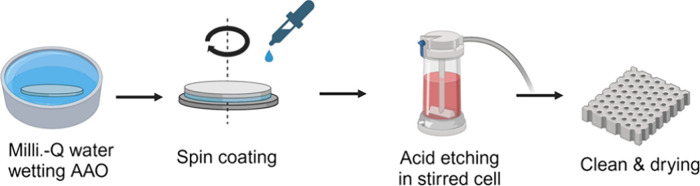
Schematic diagram of the fabrication methodology for the BCP membranes TFC used in this work.

The fabrication method employed in this work is illustrated in Figure 1. First, AAO microfiltration discs, with a rated pore size of 0.2 μm and a diameter of 25 mm, were wetted in Milli-Q water. They were then placed on a 3 cm *×* 3 cm plasma-cleaned silicon wafer. Prior to coating the BCP solution, a spinning test was conducted to evaluate the adhesion between the AAO and the silicon substrate. The AAO disc should remain firmly attached to the silicon wafer surface at rotational speeds of up to 5000 rpm. This adhesion is attributed to the water filling the interspace between the two materials. Furthermore, wetting the AAO samples prevented the PS-*b*-PMMA solution from penetrating the pores during the spin-coating step, as both PS-*b*-PMMA and toluene are immiscible with water. Next, a 4 wt % solution of PS-*b*-PMMA in toluene was prepared and filtered through a 0.45 μm PTFE syringe filter. The polymeric solution was then uniformly spread over the entire surface of the AAO disc, followed by spinning at 1000 rpm for 60 s. After completing the spin-coating process, the membrane sample was carefully detached from the silicon wafer substrate using tweezers, holding it by the plastic O-ring area to avoid damaging the brittle AAO discs. Selective removal of the PMMA block was carried out by exposing the membrane sample to UV irradiation (Vilver, France, λ= 254 nm) for 10 min to induce PMMA degradation via unzipping or chain scission of the MMA bonds. Following UV treatment, the sample was transferred to an Amicon stirred cell, where acetic acid was used to dissolve and remove the resulting MMA fragments. The complete removal and subsequent formation of the porous membrane was achieved by continuing the acetic acid treatment until a steady flux was observed. Finally, the sample was flushed with Milli-Q water and left to dry at room temperature overnight, for subsequent SEM and AFM characterization.

### 2.3 Surface Characterization of PS-*b*-PMMA TFC Membranes

Atomic Force Microscopy (AFM) was performed using a Bruker Dimension Icon instrument equipped with ScanAsyst-Air probes (Bruker). Postprocessing and image analysis were done using WSxM software.^45^ For SEM measurements, a Quorum SC7620 sputter coater was used to deposit a thin layer of gold to improve imaging quality. The SEM imaging was carried out with a Carl Zeiss XB1540 Cross-Beam system. The instrument was operated at a voltage of 10 kV with an SE2 signal source, resulting in imaging magnifications ranging from 13,000x to 16,000x.

### 2.4 Ultrafiltration

The experimental setups and calculations for the selectivity and permeability tests are illustrated in Figure S1 (Supporting Information). Twenty-five mm diameter membrane candidates were loaded into the Amicon stirred cell (model 8010), sourced from Merck Millipore. A Thermo Fisher UV–vis Spectrometer G10S, Shimadzu refractive index detector, and a Shimadzu RF-6000 spectro-fluorophotometer were used to measure the concentration of feed and permeate. In all cases, filtration was terminated after sufficient volume of permeate was collected, normally 20 L/m^2^ (liters of permeate per membrane area).

### 2.5 Size Exclusion Chromatography (SEC)

A Shimadzu 2030 HPLC system, along with a TSKgel G5000PWXL (08023) size exclusion chromatography column from Tosoh, was used to fractionate dextran of different molecular weights. A refractive index detector (RID-20A from Shimadzu) was used to measure the concentration of dextran. The column was calibrated with monodisperse dextran standards (Sigma-Aldrich) of varying molecular weights to correlate retention time with molecular weight. The mobile phase was 1 *×* PBS buffer running at 0.3 mL/min due to the pressure restrictions of the size exclusion column.

## 3 Results and Discussion

### 3.1 Fabrication of PS-*b*-PMMA TFC Membranes

**Figure 2:**
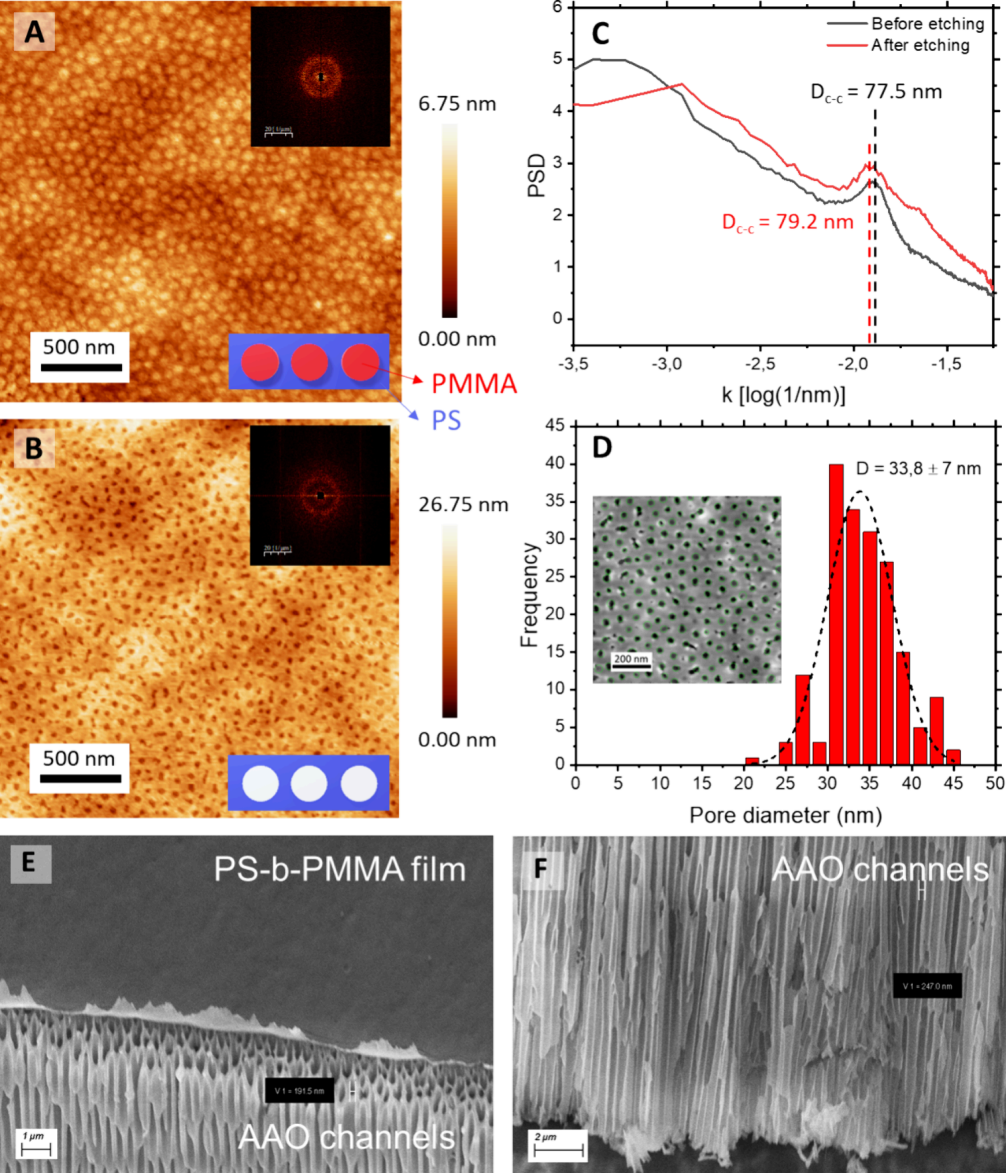
(A) AFM micrograph of the PS-*b*-PMMA film deposited on top of the AAO disk. (B) Topographical AFM micrograph of the PS porous film created after PMMA selective removal by UV/acetic acid treatment. (C) PSD of corresponding topographical AFM images. (D) Pore size distribution of the porous PS membrane obtained by image analysis of the corresponding AFM image. (E) Cross-sectional SEM image of the etched PS-*b*-PMMA TFC membrane overlying the porous AAO structure. (F) Cross-sectional SEM image showing the channels within the AAO structure.

The fabrication methodology followed in this work is illustrated in Figure 1. The process begins with the spin-coating of the BCP solution onto the AAO membrane. To do that, a flat rigid surface is required in order to support the AAO membrane and ensure uniform coating of the polymeric solution. As shown in Figure S2, this was achieved by attaching the fully wetted AAO microfiltration disc to a plasma-cleaned silicon wafer. Thus, water adhesive forces were enough to hold the AAO membrane in place, even at rotational speeds of up to 5000 rpm. Subsequently, the PS-*b*-PMMA solution was deposited onto the attached AAO disk at 1000 rpm. Ellipsometric measurements confirmed the formation of a homogeneous BCP film with a thickness of around 150 nm. Figure 2A shows the AFM topographical micrograph obtained immediately after the spin-coating process. The results indicate the successful formation of a hexagonal cylindrical structure, which is primarily dictated by the volume fraction of each block in the BCP used, which in this case was 70:30 PS: PMMA. As a result, a structure of PMMA cylinders embedded in a PS matrix is expected. The topographical profile shown in Figure S3B reveals small bumps with heights of approximately 1 nm, corresponding to the PMMA cylinders. This observation confirms the cylindrical morphology, as the alternative possibility—hexagonal micelle assembly—would exhibit significantly greater height variations. Regarding orientation, the AFM micrograph confirms an out-of-plane alignment, with the PMMA cylinders oriented perpendicular to the surface of the substrate.

The orientation of BCP nanostructures, particularly PS-*b*-PMMA, has been an active area of research in recent years. Numerous strategies have been developed to control this orientation, focusing on parameters such as surface neutrality or preferential interactions,^46,47^ film thickness,^48^ and the topography of the underlying substrate,^49^ among others. In our case, the topography of the underlying AAO substrate appears to promote the perpendicular orientation of the nanostructures to the surface without the need for any subsequent annealing treatment, such as thermal or solvent annealing, even at moderate film thickness. This orientation was further enhanced by selecting a film thickness comparable to the BCP pitch.^48^

Once the structure of the BCP film was confirmed, the second step in the fabrication procedure involved the selective removal of the PMMA block to create the desired porous structure. Following the protocol detailed in the experimental section TFC membranes were exposed to acetic acid flow in the stirrer cell after being previously UV irradiated for 10 min. Figure 2B shows the AFM topographical micrograph of the TFC membrane after the acetic acid treatment, revealing a clear inversion in the observed topography. This inversion confirms the selective removal of the PMMA block. Figure S3 compares the topographical profiles, confirming this selective removal. Consequently, the PMMA regions are replaced by empty pores approximately 30 nm deep. However, due to convolution and steric effects between the AFM tip and the porous structure, the probe cannot fully penetrate into the pores, leading to an underestimation of their depth. As a result, AFM provides only superficial information about the sample’s porosity. To verify the porous nature of the BCP film and the effectiveness of the UV/acetic acid treatment in removing the PMMA block, a similar sample was prepared on a poly(vinyl alcohol) (PVA) film. Since PVA is highly water-soluble, immersing the TFC membrane in water dissolves the PVA, allowing the BCP film to detach from the substrate. This enables the backside of the film to be studied using AFM, providing a more complete assessment of the film’s porous structure. Figure S4 presents the AFM micrograph of the membrane’s backside, revealing a porous structure similar to that observed on the top side (Figure 2B). This confirms the successful removal of the PMMA block and highlights the formation of a uniform porous structure.

The stability of the BCP structure is supported by the comparison of the power spectral density profiles from both AFM micrographs, which demonstrate that the center-to-center distances before and after selective removal (Figure 2C). Image analysis of the AFM micrographs enables the determination of the pore size distribution (Figure 2D). Consequently, the pore diameter of the BCP film is established at 33 nm ±7 nm. Finally, the adhesion of the BCP film to the AAO membrane was evaluated. Since AFM is limited to characterizing relatively small surface areas (μ^2^), SEM was chosen as an alternative methodology for this analysis. As depicted in Figure 2E, the SEM top view image reveals the thin PS-*b*-PMMA layer as a homogeneous selective layer, while the long AAO channels serve as the backing layer. The edge of the PS-*b*-PMMA thin film appeared partially lifted due to the mechanical cutting. At the interface, there was no evidence of blockage in the AAO backing layer by PS-*b*-PMMA, which aligns with the objectives of this novel fabrication design. Furthermore, there were no issues of delamination or defects associated with this direct spin-coating method. The cross-sectional SEM image of the AAO membrane (Figure 2F) shows no evidence of blockage or infiltration of the BCP film into the inorganic porous structure.

### 3.2 Evaluation of Membrane Performance

#### 3.2.1 Characterization of the Feedstock Solutions

Three different systems have been utilized in this work as model molecules to evaluate the membrane’s performance and its selectivity-permeability ratio: latex nanoparticles, proteins (BSA, mAb and Tg), and FICT-dextran nanoparticles of different molecular weights (4 kDa, 40 kDa, 150 kDa, and 500 kDa, respectively). The rationale behind this selection is to characterize the membrane’s performance in three significant scenarios within bioseparation processes. Specifically, latex nanoparticles replicate the physical properties of viral products;^50,51^ proteins are representative of a highly active research field in purification; and dextran nanoparticles of varying molecular weights simulate the sizes of organic acids, monoclonal antibodies (mAbs), and mAb aggregates,^52^ while also allowing for the study of membrane permeability.^53^ Consequently, the first step in evaluating the membranes’ performance involved preparing and thoroughly characterizing the feedstock solutions of the systems previously mentioned.

**Figure 3:**
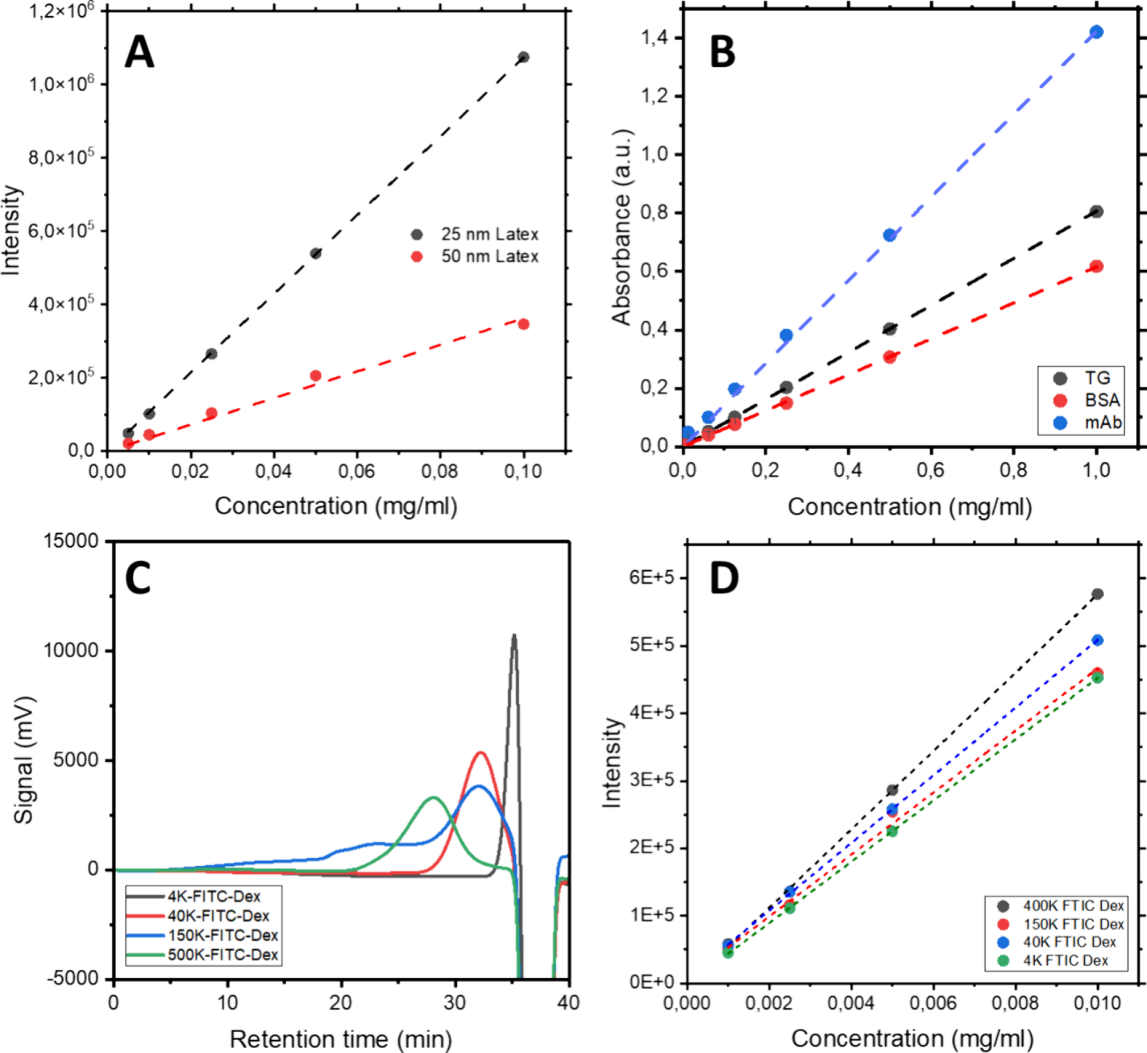
(A) Calibration curve of fluorescence intensity for 25 and 50 nm latex nanoparticles at varying concentrations (*R*^2^=0.99). (B) Standard curves for BSA, mAb, and Tg measurement at an optical absorption wavelength of 280 nm at pH 7.0 (*R*^2^=0.99). (C) SEC characterization of FITC-dextran with Mw of 4 kDa, 40 kDa, 150 kDa, and 500 kDa. (D) Calibration curves of fluorescence intensity for FITC-dextran of 4 kDa, 40 kDa, 150 kDa, 500 kDa across varying concentrations (*R*^2^=0.99).

Thus, the spherical fluorescent latex nanoparticles used in this work exhibited an excitation wavelength of 552 nm and an emission wavelength of 580 nm. Particles with diameters of 25 and 50 nm were utilized in this study. Concentrations between 0.01 g/L and 0.1 g/L were employed to avoid exceeding the upper limit of the detection range of the spectrofluorophotometer used for their analysis (Figure 3A). In the case of the proteins, feedstocks of BSA, mAb, and thyroglobulin (Tg) were prepared at 1 g/L in PBS buffer. The standard curve for all three proteins exhibited a strong linear correlation at 280 nm (*R*^2^ = 0.99, see Figure 3B). The estimated protein hydrodynamic diameters were established at 7, 11, and 17 nm respectively following references shown in Table S1.

Finally, the size distribution of the fluorescein isothiocyanate (FITC)-dextran particles was assessed via SEC. Thus, particles with larger average molecular weights exhibit greater dispersion. Among these, the 150 kDa FITC-dextran displays a broader size distribution compared to the other molecules. The overlapping negative peaks observed in all samples at approximately 36 min likely result from additives or small sugar molecules, such as glucose, present in the dextran samples (Figure 3C). Since FITC-dextran is not sensitive to UV/vis detection, its intensity was measured using a Shimadzu spectrofluorophotometer. Figure 3D demonstrates a strong linear relationship between signal intensity and concentration. However, the detectable concentration of FITC-dextran ranges from 0.001 mg/mL to 0.01 mg/mL. Higher concentrations produce stronger signals that exceed the upper limit of the spectrofluorophotometer.

#### 3.2.2 Evaluation of Water Permeability

**Figure 4:**
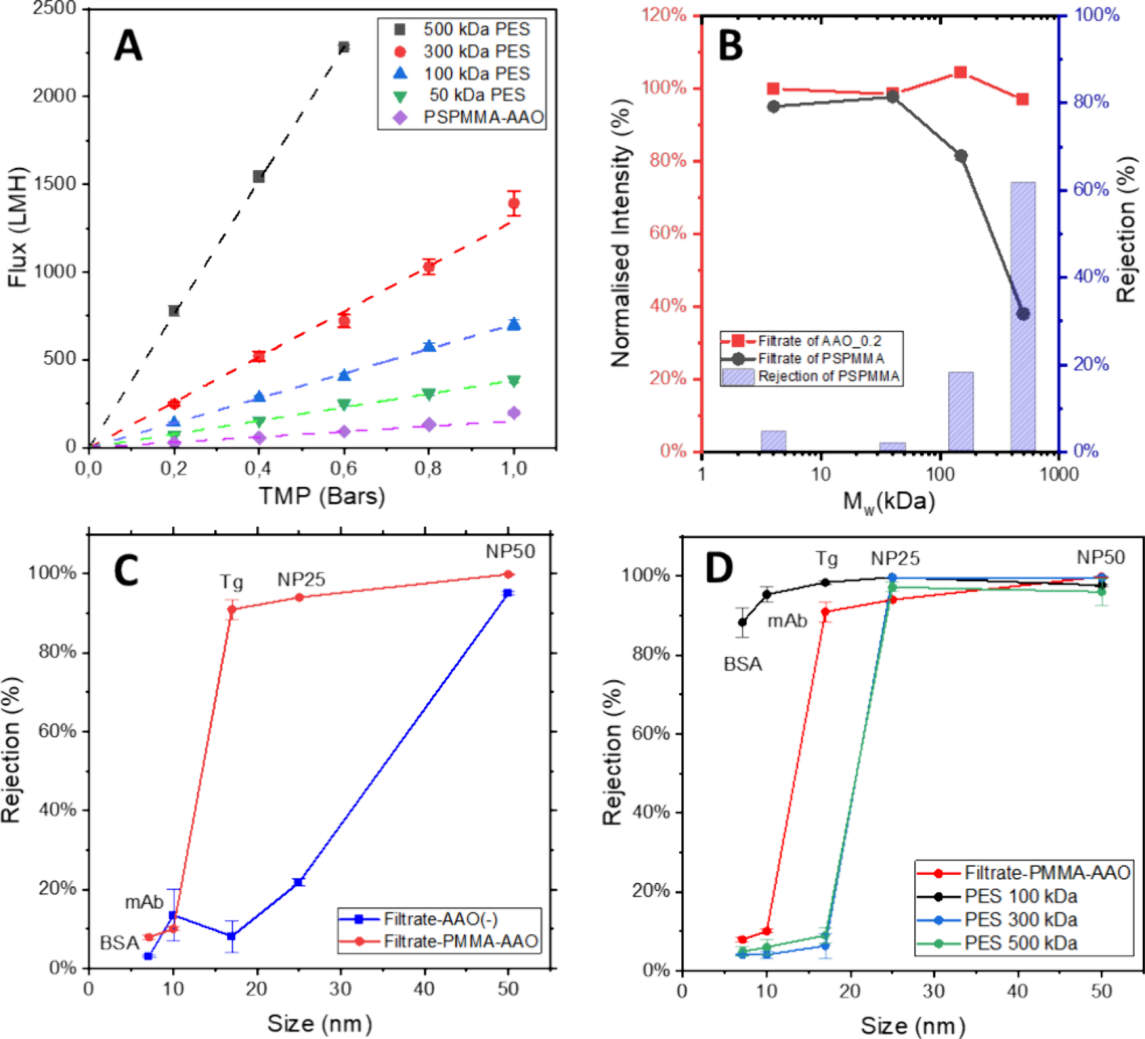
(A) Permeability of PS-*b*-PMMA TFC membrane, benchmarked with commercial 50 kDa, 100 kDa, 300 kDa and 500 kDa PES membranes. (B) Rejection rate of FITC-dextran of different molecular weights for PS-*b*-PMMA TFC membrane (black) and pristine AAO discs (red). (C) Rejection rate of PS-*b*-PMMA TFC membranes, tested with BSA, mAb, Tg, and latex nanoparticles of 25 and 50 nm diameter, benchmarked with pristine AAO discs. (D) Rejection rate of a PS-*b*-PMMA TFC membrane, compared with commercial PES 100, 300, and 500 kDa membranes.

The next step in evaluating the performance of the fabricated TFC membrane was to confirm its water permeability. This is crucial not only for verifying the membrane’s applicability, as bioseparation processes typically involve aqueous media, but also to ensure the complete formation of the BCP porous structure. Figure 4A presents the water permeability data for the PS-*b*-PMMA TFC membrane developed in this study, alongside a series of Biomax commercial PES membranes used for comparison. The corresponding error bars represent the data from three replicates, ensuring the accuracy and reliability of the observations. For all studied membranes, the relationship between water flux (LMH) and transmembrane pressure (bars) exhibited a strong linear fit (R^2^ = 0.99). The water permeability test of the PS-*b*-PMMA TFC membrane confirmed the successful formation of a fully porous structure during the UV/acetic acid treatment. To further validate this observation, the backside of the BCP film was analyzed using AFM. Figure S4 presents the corresponding topographical AFM micrograph, revealing a porous structure similar to that observed on the top side of the membrane (Figure 2B).

However, the water permeability value of the PS-*b*-PMMA TFC membrane (154 ± 9.2 LMH/bars) appears to be significantly lower than that of its more comparable PES counterpart (50 kDa PES membranes, 383 ± 9.68 LMH/bars). This discrepancy could be attributed to two factors: first, incomplete PMMA removal, and second, the hydrophobic nature of the PS porous layer. The former was discarded, as extending the UV/acetic acid treatment did not lead to any improvements in water permeability. The latter is an intrinsic issue with membranes based on PS BCPs, which are known to exhibit lower permeability due to their hydrophobic nature. However, since this does not affect membrane selectivity it is not a critical factor for the applicability of the created membrane.

#### 3.2.3 Evaluation of Membrane Rejection

As detailed in the experimental section, the selectivity of the BCP-AAO TFC membranes was evaluated using an Amicon stirred cell. Similar to the water permeability test, TFC performance was compared with 100, 300, and 500 kDa PES membranes as positive controls, and 0.2 μm AAO discs as negative controls, with each condition tested in triplicate.

##### 3.2.3.1 Selectivity toward FITC-Dextran Nanoparticles

To quantify the rejection rate of the membranes, the fluorescence of known concentrations of FITC-dextran in the feedstock was measured before and after the filtration experiment. A pristine AAO disc was used as a control sample to assess the effect of the active BCP layer in this study. Figure 4B shows the normalized fluorescence intensity of the permeate (filtrate) of FITC-dextran nanoparticles compared to the original feedstock for both the PS-*b*-PMMA TFC membrane and the AAO disc (backing material alone), also known as the sieving coefficient. In contrast to the original AAO disk, the PS-*b*-PMMA TFC membrane achieved over 60% rejection at 500 kDa, while smaller particles (4 kDa and 40 kDa) were not retained by the membrane. This highlights the superior performance of the TFC membrane compared to the commercial AAO disc, demonstrating size-based selectivity due to the incorporation of the porous BCP film. However, the molecular weight of FITC-dextran cannot cover the molecular weight cutoff (MWCO) of the membrane candidate, which is defined as 90% of the feed being rejected. Consequently, further sieving experiments involving proteins and latex NPs were conducted to screen the membrane candidates’ selectivity.

##### 3.2.3.2 Selectivity toward Proteins and Latex Nanoparticles

As an initial approach, and following the methodology used in the previous section, membrane selectivity was tested with various proteins (BSA, mAb, and Tg) alongside 25 and 50 nm diameter latex nanoparticles. To obtain comparable results to those presented in Figure 4B, the findings were compared against those from a pristine AAO disc. Figure 4C shows the rejection profile of both AAO and TFC membranes. Thus, while the AAO discs (shown in blue) displayed a gradual increase in the rejection rate, ranging from 10% rejection for BSA, mAb, and Tg, to over 90% rejection for 50 nm latex nanoparticles, the rejection profile of the PS-*b*-PMMA TFC membranes (shown in red) was significantly sharper, demonstrating over 90% rejection of Tg and latex nanoparticles, while retaining less than 10% of mAb and BSA. Notably, the PS-*b*-PMMA TFC membrane achieved more than 95% rejection of 25 nm latex nanoparticles and over 99% rejection of 50 nm latex nanoparticles, despite the observed average surface pore size of the membrane being approximately 38 nm.

Subsequently, the performance of the TFC membrane was evaluated against PES standard membranes with different pore sizes (100, 300, and 500 kDa) using the same model molecules. Figure 4D shows the rejection profile of the PES membranes. The 300 kDa and 500 kDa membranes showed similar rejection profiles, with over 90% of BSA, mAb, and Tg passing through both membranes, while over 95% of 25 and 50 nm latex nanoparticles were rejected. In contrast, the 100 kDa PES membranes showed over 90% rejection for all three proteins and latex nanoparticles.

A comparison of these results with those of the TFC membranes is also presented in Figure 4D. Thus, while the commercial PES membranes were unable to separate proteins of different sizes, likely due to their broader pore sizes distribution, the PS-*b*-PMMA TFC membranes effectively differentiate between mAbs and Tg based on molecular weight. This observation further suggests that the MWCO of the PS-*b*-PMMA TFC membrane ranges between 100 kDa and 300 kDa.

**Figure 5:**
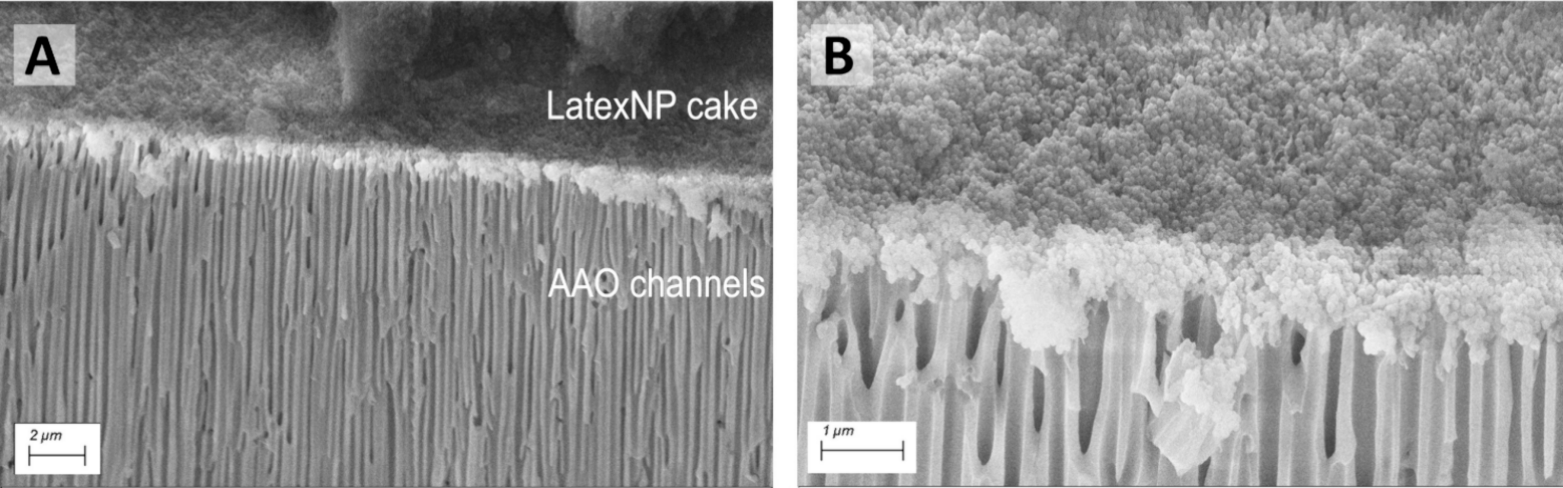
SEM imaging of a PS-*b*-PMMA TFC membrane after filtering 100 nm latex NPs. (A) Cross-sectional view showing the latex NP cake layer on top of the anodic aluminum oxide (AAO) channels. (B) Close-up of the interface, highlighting the morphology and distribution of the latex NP aggregates within the AAO structure.

As a final experiment, and to demonstrate the membrane’s capacity to effectively retain the filtered feedstock, SEM was conducted on PS-*b*-PMMA TFC membranes that had filtered 100 nm latex nanoparticles, as these nanoparticles were large enough to be visualized. The postfiltration SEM images shown in Figure 5**A-B** demonstrate the accumulation of the latex NPs in the form of a dense cake layer on the membrane’s top surface, while the AAO channels remain free of latex nanoparticles. This demonstrates that the PS-*b*-PMMA TFC membrane can effectively reject and retain the filtered species, suggesting potential applications for the retention or removal of larger viruses.

## 4 Conclusions

This study successfully fabricated and evaluated PS-*b*-PMMA TFC membranes using a novel spin-coating methodology on AAO substrates. The fabrication process involved spin-coating BCP solutions, followed by selective PMMA removal with UV/acetic acid treatment, resulting in a porous membrane with an average pore size of around 33 nm. Surface characterization by AFM and SEM demonstrated the formation of a homogeneous BCP porous film on top of the AAO disc, with no potential defects, pore blockages or delamination issues. The performance evaluation demonstrated that the TFC membranes achieved rejection rates over 90% for latex nanoparticles and proteins like Tg, while exhibiting less than 10% retention for monoclonal antibodies. This notable selectivity highlights the membranes’ capability to differentiate between molecules based on size, outperforming conventional PES membranes. SEM analysis further confirmed the membrane’s performance, demonstrating its ability to retain filtered species. Overall, the PS-*b*-PMMA TFC membranes exhibit a remarkable combination of structural integrity, permeability, and selectivity, making them promising candidates for various bioseparation processes. Moreover, the methodology presented here provides a valuable framework for the rapid screening of BCP-based membranes.

## Supporting Information Available

Ultrafiltration setup and performance evaluation; AFM characterization of block copolymer membranes; protein concentration determination using Beer–Lambert’s law; hydrodynamic size estimations of model proteins; supplementary references (PDF)
