# Supplementary material for: A Versatile Fabrication Route for Screening of Block Copolymer Membranes in Bioprocessing
Source: ACS Omega. 2025 Feb 20;10(8):8630–9. doi: 10.1021/acsomega.4c11269 (PMC11886646; doi:10.1021/acsomega.4c11269)
Supplement: Supplementary file 1 — ao4c11269_si_001.pdf [file ao4c11269_si_001.pdf]

-Supporting Information-

A Versatile Fabrication Route for Screening of Block Copolymer Membranes in Bioprocessing

Ke Meng,[1,2,†] Alberto Alvarez-Fernandez,[3,†] Stefan Guldin,[2,4,5,*] and Daniel G. Bracewell[1,*]

[1] *University College London, Department of Biochemical Engineering, Gower Street, London, WC1E 6BT, UK*

[2] *University College London, Department of Chemical Engineering, Torrington Place, London, WC1E 7HB, UK*

[3] *Centro de Fisica de Materiales (CFM)(CSICUPV/EHU)-Materials Physics Center (MPC), 20018 San Sebastian, Spain*

[4] *Technical University of Munich, Department of Life Science Engineering, Gregor Mendel-Straße 4, 85354 Freising, Germany*

[5] *TUMCREATE, 1 CREATE Way, #10-02 CREATE Tower, 138602, Singapore*

[†] Authors contributed equally to this work.

E-mail: guldin@tum.de; d.bracewell@ucl.ac.uk

Ultrafiltration Setups

The experimental setups for the selectivity and permeability tests are illustrated in the following diagram (**Figure S1**). The driving force was provided by the pressurised nitrogen and controlled by a pressure regulator. The gas pushed the feedstock from the reservoir into the stirred cell, and permeate was collected consequently. At the chosen pressure, the selectivity, measured by the sieving coefficient (S) is expressed as the ratio of the concentration in the filtrate or permeate (Cf) to the concentration in the feed (CF), shown in **Equation S1**. The solute rejection R(%) and the water permeability (P) are calculated by **Equations S2** and **S3**, where V, S, t, δP are filtration volume, effective membrane area, filtration time, and pressure difference, respectively.[1]

$$S = \frac{C_f}{C_F} \tag{S1}$$

$$R\ (\%) = \left(1 - \frac{C_f}{C_F}\right) x\ 100 \tag{S2}$$

$$P(LMH * bar^{-1}) = \frac{V}{S\ x\ t\ x\ \delta P} \tag{S3}$$

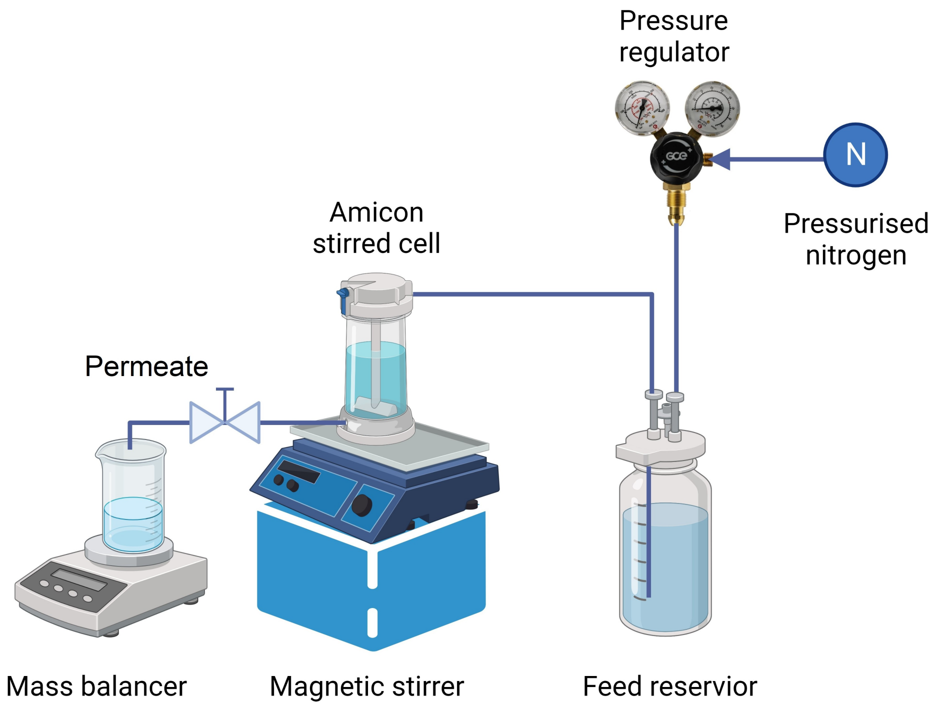

Figure S1. A schematic diagram of the Amicon stirred cell ultrafiltration set-up.[2]

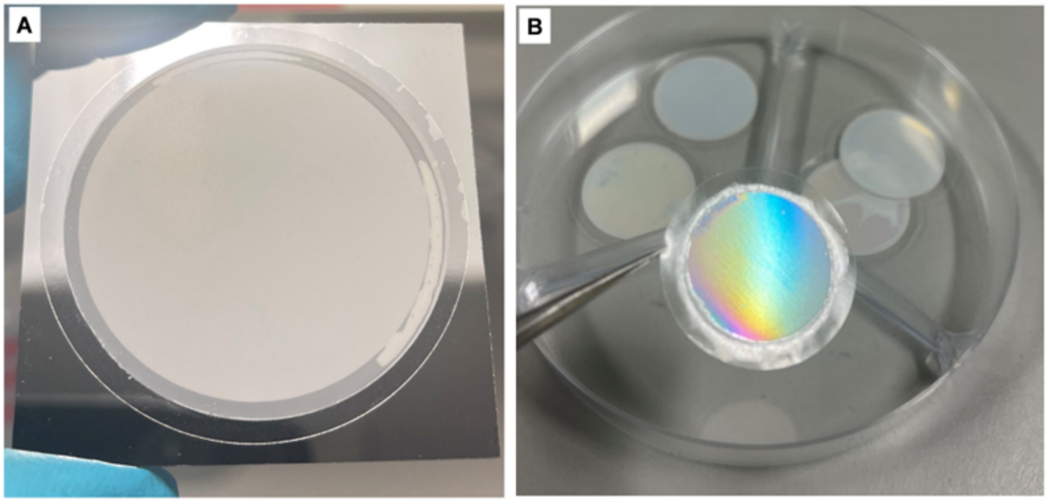

Figure S2. (A) AAO membrane attached to the rigid silicon substrate. (B) AAO disk after the deposition of the PS-b-PMMA film by spin-coating.

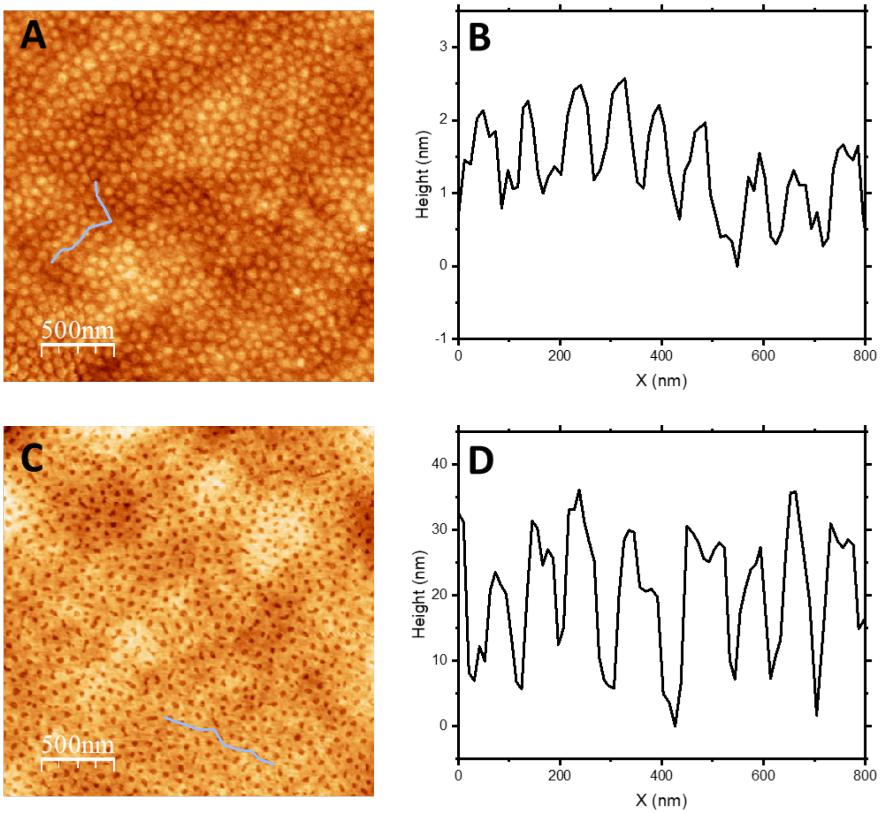

Figure S3. AFM topographical images and corresponding height profiles of the PS-b-PMMA film before (A, B) and after (C,D) etching.

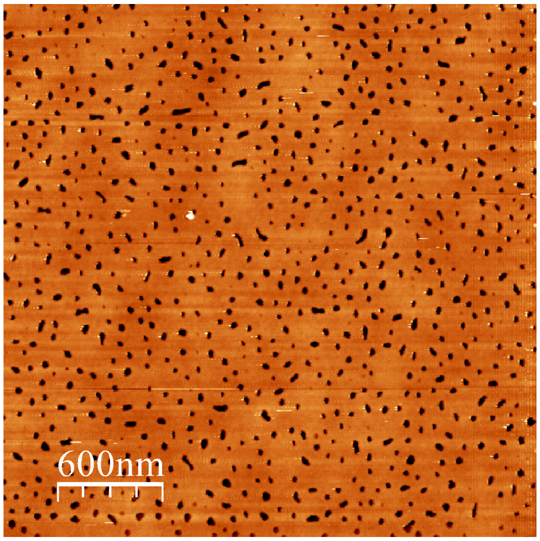

Figure S4. AFM topographical micrograph of the membrane´s back side after acetic acid etching treatment.

Calculation of protein concentration

The relationship between optical absorbance (*A*) and protein concentration (*c*) should follow Beer-Lambert's law shown in **Equation S4**, where ϵ and l represent and molar extinction coefficient and optical path length.

$$A = \epsilon \times l \times c \tag{S4}$$

Estimations on protein hydrodynamic size

Table S1. Hydrodynamic diameter estimation of model proteins: Bovine serum albumin (BSA), trastuzumab (mAb), and thyroglobulin (Tg).

Protein	Approximate Molecular Weight (kDa)	Hydrodynamic diameter (nm)	Reference
**BSA**	66.5	7	3,4
**mAb**	150	10.2-11.8	5,6
**Tg**	660	15.7-17.2	4,7

References

1. Zong, Y.; Long, Q.; Chen, L.; Samadi, A.; Luo, H.; Liang, K.; Wan, X.; Liu, F.; Chen, Y.; Zhang, Z.; Zhao, S. Dual 2D nanosheets with tunable interlayer spacing enable high- performance self-cleaning thin-film composite membrane. *Journal of Membrane Science* **2024**, *693*, 122328.

2. Aoki, S. BIORENDER. *Biorender* **2017**,

3. Yohannes, G.; Wiedmer, S. K.; Elomaa, M.; Jussila, M.; Aseyev, V.; Riekkola, M. L. Thermal aggregation of bovine serum albumin studied by asymmetrical flow field-flow fractionation. *Analytica Chimica Acta* **2010**, *675*.

4. Verde, V. L.; Dominici, P.; Astegno, A. Determination of Hydrodynamic Radius of Proteins by Size Exclusion Chromatography. *Bio-Protocol* **2017**, *7*.

5. Salinas, B. A.; Sathish, H. A.; Bishop, S. M.; Harn, N.; Carpenter, J. F.; Randolph, T. W. Understanding and modulating opalescence and viscosity in a monoclonal antibody formulation. *Journal of Pharmaceutical Sciences* **2010**, *99*.

6. Hawe, A.; Hulse, W. L.; Jiskoot, W.; Forbes, R. T. Taylor dispersion analysis comparedto dynamic light scattering for the size analysis of therapeutic peptides and proteins andtheir aggregates. *Pharmaceutical Research* **2011**, *28*.

7. Scheidt, T.; Kartanas, T.; Peter, Q.; Schneider, M. M.; Saar, K. L.; Müller, T.; Challa, P. K.; Levin, A.; Devenish, S.; Knowles, T. P. Multidimensional protein characterisation using microfluidic post-column analysis. *Lab on a Chip* **2020**, *20.*
